# Gut-immune axis and cardiovascular risk in chronic kidney disease

**DOI:** 10.1093/ckj/sfad303

**Published:** 2023-12-13

**Authors:** Felix Behrens, Hendrik Bartolomaeus, Nicola Wilck, Johannes Holle

**Affiliations:** Department of Pediatric Gastroenterology, Nephrology and Metabolic Diseases, Charité – Universitätsmedizin Berlin, Berlin, Germany; Experimental and Clinical Research Center (ECRC), a cooperation of Charité – Universitätsmedizin Berlin and Max Delbrück Center for Molecular Medicine (MDC), Berlin, Germany; German Centre for Cardiovascular Research (DZHK), Partner Site Berlin, Berlin, Germany; Institute of Physiology, Charité – Universitätsmedizin Berlin, Berlin, Germany; Experimental and Clinical Research Center (ECRC), a cooperation of Charité – Universitätsmedizin Berlin and Max Delbrück Center for Molecular Medicine (MDC), Berlin, Germany; German Centre for Cardiovascular Research (DZHK), Partner Site Berlin, Berlin, Germany; Max Delbrück Center for Molecular Medicine (MDC), Berlin, Germany; Department of Nephrology und Intensive Medical Care, Charité – Universitätsmedizin Berlin, Berlin, Germany; Experimental and Clinical Research Center (ECRC), a cooperation of Charité – Universitätsmedizin Berlin and Max Delbrück Center for Molecular Medicine (MDC), Berlin, Germany; German Centre for Cardiovascular Research (DZHK), Partner Site Berlin, Berlin, Germany; Max Delbrück Center for Molecular Medicine (MDC), Berlin, Germany; Department of Nephrology und Intensive Medical Care, Charité – Universitätsmedizin Berlin, Berlin, Germany; Department of Pediatric Gastroenterology, Nephrology and Metabolic Diseases, Charité – Universitätsmedizin Berlin, Berlin, Germany; Experimental and Clinical Research Center (ECRC), a cooperation of Charité – Universitätsmedizin Berlin and Max Delbrück Center for Molecular Medicine (MDC), Berlin, Germany; German Centre for Cardiovascular Research (DZHK), Partner Site Berlin, Berlin, Germany; Max Delbrück Center for Molecular Medicine (MDC), Berlin, Germany

**Keywords:** cardiovascular disease, chronic kidney disease, immunity, inflammation, microbiome

## Abstract

Patients with chronic kidney disease (CKD) suffer from marked cardiovascular morbidity and mortality, so lowering the cardiovascular risk is paramount to improve quality of life and survival in CKD. Manifold mechanisms are hold accountable for the development of cardiovascular disease (CVD), and recently inflammation arose as novel risk factor significantly contributing to progression of CVD. While the gut microbiome was identified as key regulator of immunity and inflammation in several disease, CKD-related microbiome-immune interaction gains increasing importance. Here, we summarize the latest knowledge on microbiome dysbiosis in CKD, subsequent changes in bacterial and host metabolism and how this drives inflammation and CVD in CKD. Moreover, we outline potential therapeutic targets along the gut-immune-cardiovascular axis that could aid the combat of CVD development and high mortality in CKD.

## CARDIOVASCULAR DISEASE IN CHRONIC KIDNEY DISEASE

Patients with CKD suffer from markedly decreased life expectancy, mainly driven by cardiovascular events, with 10–30-fold higher cardiovascular mortality than in the general population [1, 2]. Underlying causes of CKD, i.e. hypertension and diabetes, may contribute to the marked cardiovascular phenotype in CKD, but recent meta-analyses indicated CKD as an independent risk factor for cardiovascular disease (CVD) [2–4]. In line with these findings, children and adolescents with CKD, who are lacking traditional cardiovascular risk factors, are affected by CVD [5, 6] similarly composing the leading cause of death in children on dialysis [7–10].

**Figure 1:**
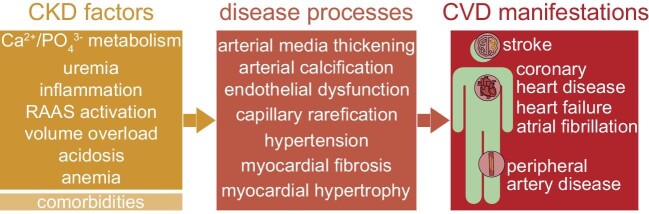
Cardiovascular disease in chronic kidney disease (CKD). Flowchart depicting CKD-related factors driving cardiovascular disease (CVD) processes that lead to a high frequency of CVD manifestations in CKD. Comorbidities refer to common conditions that coincide with CKD and also drive CVD, i.e. diabetes and hypertension. RAAS, renin–angiotensin–aldosterone system.

Patients with CKD exhibit a distinct cardiovascular phenotype and frequently suffer from left ventricular hypertrophy with myocardial fibrosis and impaired contractility, capillary rarefication, endothelial dysfunction, arterial media thickening and calcification and consequently increased arterial stiffness and atherosclerosis (Fig. 1) [2, 11]. Alterations in calcium and phosphate metabolism, sodium and volume overload, inflammation, anemia, uremia, sympathetic and renin–angiotensin–aldosterone system (RAAS) overactivation, chronic acidosis and hemostatic abnormalities are all thought to be involved in CVD development [12]. Nonetheless, therapeutic strategies improving cardiovascular outcome in CKD were of limited success so far: RAAS and SGLT2 inhibitors showed gradual success, but could not resolve the matter on their own [12]. Hence, achieving an in-depth understanding of CVD pathology and identifying new therapeutical targets remain important research areas.

In this review, we summarize the growing body of knowledge on the interaction of gut microbiome dysbiosis with the innate and adaptive immunity, promoting a chronic state of inflammation as a major risk factor for progression of CVD. Moreover, we outline novel therapeutic avenues fighting CVD with interventions targeting the microbiome and inflammation.

## INFLAMMATION IN CHRONIC KIDNEY DISEASE AND ITS CONTRIBUTION TO CARDIOVASCULAR MORBIDITY

The importance of inflammation in CVD progression was recently demonstrated by the CANTOS trial that showed marked reduction of cardiovascular events in patients with CKD that had had myocardial infarction beforehand when treated with interleukin (IL)-1β antibody canakinumab. The biggest reduction in cardiovascular events was observed when inflammation was successfully reduced by canakinumab [high-sensitivity C-reactive protein (hsCRP) <2 mg/L] highlighting the potential of reducing inflammation as a promising therapeutic strategy [13].

IL-1β composes one of the key cytokines released by pro-inflammatory macrophages [14] and while its concentrations increase in CKD, this is merely the only systemic pro-inflammatory change in CKD [15]. Patients with CKD frequently exhibit a phenotype of microinflammation as illustrated by upregulation of multiple pro-inflammatory cytokines and markers of inflammation in CKD, including IL-1β, IL-6, tumor necrosis factor (TNF)-α, transforming growth factor (TGF)-β, and hsCRP (Table 1) [15–17]. The cellular immune system is characterized by an association of monocyte counts with CKD development and progression, and monocyte subpopulations are skewed towards more non-classical and intermediate monocytes in CKD [16, 18]. T cells occur in lower numbers in CKD most likely due to decreased production and increased apoptosis [19–21] and show a pro-inflammatory pattern with decreased regulatory T cells (Tregs) with lower anti-inflammatory properties [16, 20, 22] and increased T-cell memory differentiation and senescence [20, 23–25]. Pro-inflammatory T helper 17 (T_h_17) cells were increased in CKD, and CD4^+^ and CD8^+^ T cells produced more pro-inflammatory cytokines, i.e. TNF-α and interferon (IFN)-γ [20, 22]. Pro-inflammatory changes in the T-cell phenotype correlated with markers of inflammation in CKD, specifically, Tregs correlated inversely and T _h_17 cells positively with hsCRP and IL-6 levels [22].

**Table 1:** Inflammation in chronic kidney disease (CKD).

Immune compartment	CKD-associated inflammatory changes
Soluble	↑ IL-1β, IL-6, TNF-α, TGF-β [19–21]
	↑ hsCRP [19]
Cellular	↑ total, non-classical and intermediate monocytes, ↓ classical monocytes [20, 22]
	↓ T cells, reduced egression from the thymus (↓ CD31^+^ T cells), increased apoptosis [23–25]
	↓ Tregs, lower anti-inflammatory properties [20, 24, 26]
	↑ T cell memory differentiation and senescence [24, 27–29]
	↑ T_h_17 cells [26]
	↑ IFN-γ and TNF-α production in T cells [24]

Inflammatory changes found in patients with CKD are summarized and grouped by alterations in soluble molecules and immune cell abundance and function. hsCRP, high-sensitivity C-reactive protein; IFN, interferon; IL, interleukin; TGF, transforming growth factor; T_h_17, T helper 17 cells; TNF, tumor necrosis factor; Tregs, regulatory T cells.

While CANTOS undoubtedly showed the relevance of IL-1β in CVD pathology in CKD IL-6, monocytes, monocyte-to-lymphocyte ratio, differentiated pro-inflammatory CD4^+^CD28^−^ T cells, T _h_17 cells and inversely Tregs also correlate with cardiovascular events and mortality in CKD pinpointing towards a global role of the many facets of CKD-induced inflammation in CVD pathophysiology [17, 22, 26, 27].

Reasons for inflammation in CKD are still not fully elucidated, but an increasing body of research indicates premature aging, modification of lipoproteins, oxidative stress, RAAS activation, dysregulated calcium phosphate–metabolism and, lastly, microbiome dysbiosis as driving factors [28]. Notwithstanding, the relevance of inflammation not only for CVD but also CKD progression was illustrated in the CRIC study where IL-1β, IL-6, and TNF-α associated with CKD progression [29] pinpointing towards a vicious cycle of inflammation and CKD propelling each other. In recent years, significant technical and methodological advances have especially promoted our knowledge on the impact of the microbiome on inflammation, yielding the conclusion that the microbiome is a key regulator of host immunity enabled by interaction between microbiota, microbiota-derived metabolites, and molecules on the one hand and the host mucosa, mucosa-associated, and systemic immune cells on the other hand [30, 31]. Here, we summarize the current state of knowledge on microbiome dysbiosis in CKD and subsequent alterations in microbial metabolism and its systemic relevance for inflammation and CVD that remains the main cause of death in patients with CKD.

## MICROBIOME DYSBIOSIS IN CHRONIC KIDNEY DISEASE

Under physiological conditions, microbiota contribute to homeostasis in many body functions, including immune homeostasis, which led to the established term ‘symbiosis’ [31]. In contrast, detrimental microbiota composition and microbiome effects on the host were lately termed ‘dysbiosis’ [32]. Gut microbiome dysbiosis in patients with CKD was first described by Vaziri *et al.* in 2013 [33] and ever since a growing body of work using 16S rDNA sequencing and shotgun metagenomic sequencing supplemented these first data. These studies highlighted marked changes in the taxonomic composition of the microbiome of patients with CKD [16, 34, 35]. Expansion of pathobionts (commensal bacteria usually present at low abundance but with detrimental effects when outgrowing other commensals), loss of commensal bacteria (and their beneficial metabolites and protective effects against infection) and as a consequence loss of microbiota diversity are key features of dysbiosis in several diseases [36]. When looking at the CKD microbiome, all of these key features can be observed: changes in microbiota composition with (i) reduced α-diversity; (ii) outgrowth of Enterobacteriaceae as paradigmatic example of pathobiont expansion; and (iii) loss of commensals illustrated by reduced Firmicutes abundance on phylum level [16, 33–35, 37–40]. The reasons for dysbiosis in CKD are not entirely uncovered, yet multiple factors are anticipated to contribute to dysbiosis and proteolytic fermentation in CKD including low fiber diet, muscle wasting, drug intake, uremia and constipation [41]. However, drawing conclusions from these data remains challenging due to the many bacterial phyla affected and conflicting findings that are most likely explained by the high inter-personal variability of the microbiome, especially when considering study populations from different regions. Hence, it is questionable if discoveries on differential abundance of a single bacterial species actually confer sufficient applicability to patients with CKD in other regions as the one studied. Whether changes in microbiota composition are harmful is largely dependent on their compound effect on the host, including features like fermentation pattern and metabolic capacity.

## HOW NUTRITION, MICROBIOME DYSBIOSIS AND LEAKY GUT CONTRIBUTE TO UREMIC TOXIN ACCUMULATION

A drastic change from saccharolytic to proteolytic fermentation was illustrated by differential abundance of typical bacterial genera: increases of proteolytic bacteria such as *Fusobacterium* and *Citrobacter* [16, 34, 38] and decreases of saccharolytic bacteria like *Bifidobacterium* and *Roseburia* [16, 35, 38, 40]. Regardless of cause, a shift from saccharolytic to proteolytic fermentation entails higher concentrations of proteolytic metabolites, i.e. branched-chain fatty acids, ammonia, amines, phenols and indoles, most of which are considered detrimental, and lower concentrations of saccharolytic end products, mainly SCFAs [42]. Metabolomic analyses identified multiple pathways of microbially derived metabolites to be changed in CKD, including an accumulation of indoles [prototype: indoxyl sulfate (IS)], kynurenines (both tryptophan-derived), cresols [mainly tyrosine-derived, prototype: p-cresyl sulfate (PCS)] and trimethylamine N-oxide (TMAO; choline-/carnitine-derived) and decreased SCFA concentrations [16, 43–45]. In a landmark study, Wang *et al.* could show clear associations of altered microbial metabolic functions with the fecal and serum metabolome: the microbial abundance of processes of aromatic amino acid degradation, bile acid metabolism, and SCFA synthesis were clearly associated with fecal and serum concentrations of the respective metabolites [38]. This could be mechanistically confirmed by fecal microbiota transfer (FMT) from patients with CKD to subtotally nephrectomized rodents which caused elevated IS and PCS levels when compared with rodents with CKD receiving healthy microbiome [38]. In CKD, multiple factors subsequently aggravate the metabolite imbalance and its systemic consequences in CKD.

### Nutrition

Initially, saccharolytic and proteolytic fermentation were discovered as a consequence of different dietary regimens: plant-based diet induces saccharolytic fermentation and animal products proteolytic fermentation [46]. Low-fiber diet was traditionally used to limit potassium intake in CKD but is increasingly recognized as a risk factor for dysbiosis, promoting a shift from saccharolytic to proteolytic fermentation by direct induction of proteolytic bacteria via increased substrate availability [41, 47]. Moreover, indirect mechanisms including posttranslational modification of bacterial enzymes can aggravate the metabolic imbalance, like modification of tryptophanase by sulfur-containing amino acids increasing microbial indole production and subsequently worsening kidney function [48]. Due to the association of CKD with lifestyle-associated diseases it is also conceivable that other effects observed in the classic western diet can be applied to CKD, i.e. detrimental effects of high salt intake that was shown to drive dysbiosis and lower production of the anti-inflammatory tryptophan metabolite indole-3-lactic acid (ILA) by decreasing *Lactobacillus* abundance [49]. Albeit diet is still considered the most important influence on the microbial fermentation and subsequent metabolite concentrations, a growing body of evidence indicates that baseline microbiome composition, host metabolism and other host factors like sex may largely influence the microbiome's compositional and metabolomic response to nutrition [50].

### Leaky gut

With regard to systemic effects of microbial metabolites, close attention should be paid to the local homeostasis of the intestinal mucosa and concomitant changes to intestinal barrier function. SCFAs were shown to be one of the key players in maintaining intact epithelial barrier, hence proposing that low SCFA levels could induce impaired intestinal barrier function (*leaky gut*) in CKD [42, 51] which is likely aggravated by high urea concentrations that were also shown to induce *leaky gut* [52]. Subsequently, more metabolites cross the normally tightly regulated intestinal epithelial barrier, contributing to higher systemic concentrations. Moreover, larger molecules that are physiologically not able to pass the intestinal barrier, i.e. bacterial endotoxins like lipopolysaccharide (LPS), are now able to enter the systemic circulation [42]. Indeed, plasma biomarkers markers of *leaky gut* were shown to be elevated in CKD, in particular zonulin [53] and soluble CD14 [16]. Circulating zonulin is a common biomarker of *leaky gut* and locally induces loss of epithelial tight junctions, hence actively contributing to epithelial permeability [54]. Soluble CD14 is a marker of monocyte activation, frequently released upon LPS stimulus [55] that correlates with LPS concentrations *in vivo* [56]; thus, its higher concentrations in patients with CKD indicate *leaky gut*.

### Decreased urinary excretion

Moreover, for the effects of microbial metabolites, CKD's main characteristic, decreased kidney function, also plays a decisive role and systemic concentrations of specific microbial metabolites are further elevated due to limited excretion by the kidneys as depicted by Gryp *et al.* who reported that plasma levels of indoles and cresols are primarily dependent on kidney function [57]. Indeed, multiple substances that are now considered microbial metabolites were first discovered in CKD as uremic toxins and their high concentrations were expected to be caused simply by reduced excretion through the kidneys, prominently IS and PCS as prototypic uremic toxins [58, 59]. However, Wang *et al.* demonstrated a mechanistic link between microbial production and the increased concentrations of proteolytic fermentation products in patients with CKD. Transferring fecal microbiota from patients with CKD into rodents led to increased systemic metabolite levels compared to transfers from healthy controls [37]. These findings lead to the conclusion that microbial production plays a significant role for the accumulation of metabolites in CKD. Lastly, the reduction of saccharolytic bacterial metabolites (SCFAs) in CKD cannot be attributed to kidney function and supports the important role of the altered gut microbial metabolism for bacterial metabolite concentrations in CKD.

## ALTERED MICROBIAL METABOLISM DRIVES INFLAMMATION IN CHRONIC KIDNEY DISEASE

With regard to the impact of altered microbial metabolism on host immunity in CKD, direct effects on immune cells must be separated from indirect effects that mainly include effects on the local homeostasis of the intestinal mucosa. Considering the latter, the aforementioned *leaky gut* phenotype may drive monocyte activation via elevated systemic LPS concentrations [42]. Such effects may also be aggravated by increased microbial LPS production due to the shift towards more Proteobacteria and higher immunogenicity of Proteobacteria-derived LPS when compared to other subforms [60]. Direct effects of the microbiome on immune cells are mainly driven by systemic accumulation of microbial metabolites as depicted in the following paragraphs.

### Amino acid-derived metabolites

Multiple circulating microbial metabolites with altered abundance in CKD have a profound impact on immunity and inflammation. Of tryptophan metabolites altered in CKD, the broadest knowledge on metabolite-immune interaction is present for IS that triggered pro-inflammatory cytokine release from monocytes/macrophages [TNF-α, IL-1β, IL-6, monocyte chemotactic protein 1 (MCP1)] and induced reactive oxygen species (ROS) for which different mechanisms were described, including activation of the aryl hydrocarbon receptor (AhR), induction of Notch and yes-associated protein (YAP) signalling, and β-catenin inhibition [61–63]. In endothelial and vascular smooth muscle cells (VSMCs) IS increased IL-6 release in via AhR and NF-κB (nuclear factor kappa-light-chain-enhancer of activated B cells) activation [64] which could be confirmed on tissue level in aortas of CKD rats exposed to additional high IS concentrations that showed an activation of inflammatory pathways as compared to CKD rats without additional IS [65]. In tubular epithelial cells IS induced a signal transducer and activator of transcription 3 (STAT3)-mediated pro-inflammatory (MCP1) and pro-fibrotic response [TGF-β, alpha smooth muscle actin (α-SMA)] [66] while adipocytes produced more ROS upon IS stimulus, leading to increased TNF-α and IL-6 [67]. In addition, IS has deleterious effects on CD4^+^ T cells, namely apoptosis induction and reduced proliferation, a phenotype that matches observations of reduced total T-cell and Treg counts in patients with CKD [68].

Uremic toxin PCS, being a tyrosine-/phenylalanine-derived microbial metabolite rather than tryptophan-derived, seems to confer similar pro-inflammatory effects like IS according to a smaller number of studies: PCS also induced aortic inflammation [65] and CD4^+^ T-cell apoptosis and reduced proliferation paired with mitochondrial dysfunction in these cells [68].

Apart from IS, tryptophan metabolite ILA also showed significant interaction with inflammatory processes, and despite being increased in CKD [16] had anti-inflammatory effects, indicating that products of accelerated indole pathway do not only have harmful effects but may in part actually be beneficial as already reviewed by Vanholder *et al*. previously [69]. In particular, ILA was shown to block T_h_17 differentiation [49] and inhibit inflammatory responses in macrophages (NF-κB inhibition) and intestinal epithelial cells/intestinal organoids upon LPS/IL-1β exposure (decreased IL-8 release) [70, 71]. The latter effects were mediated by AhR induction, highlighting that AhR signalling may have pleiotropic effects in CKD depending on cell type, ligand or tissue/experimental conditions with precise mechanistics of contradictory AhR effects still undiscovered [72].

### Trimethylamine N-oxide

Choline-/carnitine-derived TMAO, that gained considerable attention as a CVD biomarker in recent years, seems to have relatively specific pro-inflammatory effects limited to the vasculature. TMAO induced endothelial activation and inflammation via endoplasmic reticulum (ER) and mitochondrial stress as depicted by transcriptomic profiles [73]. Moreover, TMAO elicited NLRP3 inflammasome and NF-κB activation with subsequent IL-1β release in endothelial cells, VSMCs and mouse aortas [74, 75]. In VSMCs, TMAO upregulated vascular cell adhesion molecule 1 (VCAM-1) and thereby increased macrophage recruitment to vascular lesions [76].

### Short-chain fatty acids

Lastly, SCFAs, most relevantly acetate (C2), propionate (C3), and butyrate (C4), have well-described effects on immune cells and inflammation. Most commonly known, SCFAs confer anti-inflammatory, homeostatic effects by induction of Tregs [77, 78]. This is supplemented by reduced production of pro-inflammatory IL-17 in γδ T cells [79] and increased release of anti-inflammatory IL-22 from CD4^+^ T cells and innate lymphoid cells [80]. More recent studies also highlighted that regulatory B cells are similarly promoted by SCFAs [81, 82], yet, the effects on B cells were mediated by different mechanisms: anti-inflammatory effects on T cells are likely mediated by GPR41/43 activation and histone deacetylase inhibition [77–80] while B cells were influenced via metabolic changes and SCFA-induced increased microbial indole acetate production and subsequent AhR activation in B cells [81, 82]. While all of the above-mentioned SCFAs (acetate, butyrate, propionate) contribute to these anti-inflammatory effects, butyrate was recently proven to also promote immune surveillance via CD8^+^ cytotoxic T cells and macrophages. Butyrate boosted CD8^+^ T-cell anti-tumor activity by promoting IL-12 signalling in mice and correlated with chemotherapy response in patients with gastrointestinal cancer [83]. Furthermore, butyrate increased the memory potential of CD8^+^ T cells by changing their metabolic profile allowing long-term survival via fatty acid-skewed metabolism [84]. Monocyte-to-macrophage differentiation in the presence of butyrate led to higher macrophage antimicrobial activity mediated by a cascade of altered metabolism with decreased glycolysis, increased AMP levels, inhibited mTOR signalling and finally decreased macrophage autophagy and increased ROS production. Butyrate also induced histone deacetylase inhibition in macrophages—as known from T cells—increasing release of anti-microbial proteins like calprotectin. In mice, butyrate supplementation decreased susceptibility to oral *Salmonella* and *Citrobacter* infection, yet, it remains unclear if this protective effect was solely mediated via macrophages or whether beneficial effects on the microbiome and T cells also contributed to alleviated infection [85]. In aggregate, lower SCFA levels in CKD likely dampen the beneficial effects of SCFAs on the immune system, leading to reduced regulatory immune function and impaired immune surveillance. Both features were indeed observed in CKD, as depicted by the several aforementioned studies on Treg function in CKD and the markedly increased susceptibility to infection in CKD [86].

## GUT–IMMUNE–CARDIOVASCULAR AXIS IN CHRONIC KIDNEY DISEASE

**Figure 2:**
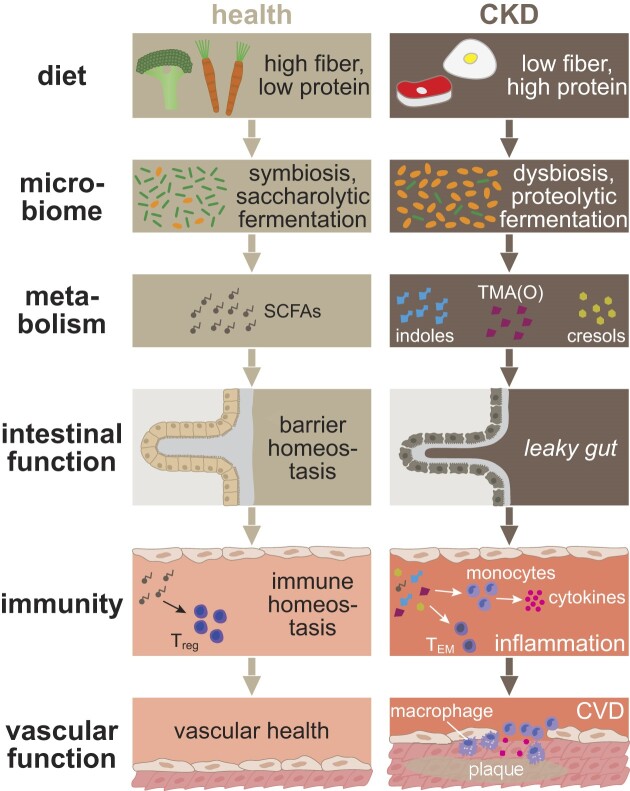
Gut-immune-cardiovascular axis in chronic kidney disease (CKD). Changes in diet, gut microbiome and metabolism drive cardiovascular disease (CVD) in CKD via dysregulation of intestinal function and inflammation. T_EM_, effector memory T cell; TMA(O), trimethylamine (N-oxide); T_reg_, regulatory T cell.

The previous chapters highlighted that inflammation, promoted by microbiome dysbiosis, is a common feature in CKD that associates with cardiovascular morbidity (summarized in Fig. 2). Supported by phenotypical overlaps in microbiome-immune interactions (e.g. dysbiosis leading to lower SCFAs and subsequently decreased Treg induction) and associations of immunity with CVD (e.g. decreased Tregs associated with CVD) a mechanistic link between the microbiome, immunity/inflammation and CVD seems probable in CKD. The homeostatic importance of the microbiome-immune axis for cardiovascular health was demonstrated in mice lacking gut microbiome (germ-free mice or antibiotic depletion) for chronic and acute cardiovascular conditions. Hypertensive organ damage in both heart and kidney was aggravated in germ-free mice associated with low abundance of SCFAs leading to pro-inflammatory changes, i.e. increased T_h_17 differentiation [87]. After experimental myocardial infarction (MI), the microbiome was indispensable for response to injury and post-MI recovery via myeloid immune response. Microbiome depletion led to a lack of SCFAs resulting in insufficient myeloid immune response, consequently increasing post-MI mortality [88].

In CKD, high fiber intake (generally facilitating symbiosis and consequently anti-inflammatory effects) associated with favorable cardiovascular outcome in cohort studies. Dietary protein-to-fiber ratio correlated with cardiovascular morbidity [89], and all-cause and cardiovascular mortality in CKD were ∼40% lower in the highest quintile of dietary fiber intake as compared to the lowest quintile [90]. In a recent preprint, high-fiber (inulin) diet altered the microbiota in CKD rats increasing *Bifidobacterium* and *Lactobacillus* while decreasing Clostridiaceae and Ruminococcaceae. This was accompanied by decreased levels of IS, PCS, and TMAO and attenuated aortic calcification, left ventricular hypertrophy and cardiac fibrosis markers (TGF-β) [91]. High fiber also induced increased SCFA levels alleviating hypertension, cardiac hypertrophy and perivascular fibrosis via GPR41, GPR43, and GPR109A activation in hypertensive mice [92].

On metabolite level, CKD-associated changes, namely increases in IS, TMAO, and PCS and decreases in SCFAs, markedly promote CVD. IS correlated with carotid intima-media thickness, left ventricular mass and worsening of pulse wave velocity in children with CKD [93]. IS and PCS promoted vascular calcification in mice via activation of inflammation and coagulation pathways in vascular tissue [65]. IS-induced Notch signalling in macrophages was shown to be functionally involved in driving atherosclerosis as Notch inhibition lowered IS-induced vascular phenotype and IL-1β expression in mice, which was translatable to CKD mice without additional IS supplementation where Notch inhibition lowered aortic plaque size and brachiocephalic artery stenosis [61].

TMAO clearly associated with CVD in cohort studies with TMAO levels highly dependent on choline intake and presence of choline-metabolizing bacteria in the microbiome [94]. High-choline diet induced atherosclerosis in mice in a microbiome-dependent manner via TMAO-dependent inhibition of reverse cholesterol transport in macrophages [95, 96] and monocyte/macrophage-derived osteopontin [97]. In the vasculature, TMAO directly promoted vascular cell adhesion molecule 1 (VCAM-1) upregulation in VSMCs, thereby increasing macrophage recruitment to vascular lesions [76]. TMAO also led to osteogenic response in aortic valve interstitial cells via ER and mitochondrial stress and subsequent NF-κB activation causing increased aortic valve thickness in mice [98]. While none of these TMAO studies were performed specifically in patients with CKD/animal models, high TMAO levels and cardiovascular burden in CKD lend credence to the applicability of these mechanisms to CKD.

SCFAs exhibited considerable potential in ameliorating CVD in animal models of hypertension and atherosclerosis. Propionate had beneficial effects on all levels of the gut-immune-cardiovascular axis, including reduced dysbiosis (increased α-diversity and decreased proteobacteria), *leaky gut* (decreased plasma LPS), systemic, vascular, and cardiac inflammation (decreased systemic TNF-α, IL-1β, IL-6, and vascular and cardiac T cell and macrophage infiltration), vascular calcification and cardiac remodeling (LV hypertrophy and fibrosis) [99, 100]. One of the studies could confirm that these effects are mediated to a large portion by promotion of Tregs through propionate [100]. Low SCFA levels in CKD paired with associations of a pro-inflammatory T-cell phenotype with CVD in CKD and evidence that ablation of T cells in CKD mice improves cardiac phenotype [24] give rise to the idea that a cascade of dysbiosis, reduced SCFAs and, subsequently, a pro-inflammatory T-cell phenotype could play a pivotal role in CKD-CVD.

## TARGETING MICROBIOME-DRIVEN INFLAMMATION FOR CARDIOVASCULAR DISEASE PREVENTION IN CHRONIC KIDNEY DISEASE

**Figure 3:**
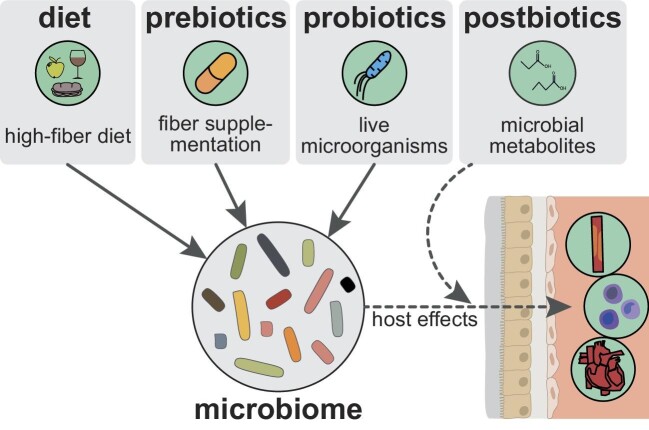
Microbiome-targeted therapy of cardiovascular disease (CVD). Dietary modifications, prebiotics and probiotics beneficially influence the microbiome and may form promising candidates to tackle dysbiosis-driven inflammation and subsequent CVD in CKD. Postbiotics, such as short-chain fatty acids, supplement beneficial microbial metabolites, likewise with positive effects on intestinal barrier function (leaky gut) and inflammation. All microbiome-targeted therapeutic strategies aim to improve inflammatory status and cardiovascular health, which is displayed schematically on the right.

Research on microbiome-targeted and anti-inflammatory therapeutic approaches in CVD gained traction in recent years. The microbiome can be targeted by dietary interventions like high-fiber diet and different -biotics designed to have beneficial effects on the host, which include prebiotics (nonviable alimentary substances modulating the microbiome) [101], probiotics (live microorganisms) [102], postbiotics (bacterial metabolic products) [103], and synbiotics (combination of pre- and probiotics) (Fig. 3) [104]. As outlined above, high-fiber diet is beneficial for cardiovascular health, yet, its efficacy as a therapeutical option in CKD remains to be tested, but a randomized controlled trial is under way analysing the influence of high-fiber diet on microbiome, inflammation, and uremic solutes in CKD [105]. Notwithstanding, in case of favorable results further trials with bigger cohorts, longer duration and cardiovascular endpoints are needed. Probiotics also seem a promising approach to lower CVD burden in CKD as probiotic intake was associated with lower inflammation in patients with CKD [106]. *Lactobacillus* improved kidney function and fibrosis in CKD mice [107], and, even though its cardiovascular effects have not been shown in CKD yet, it improved post-MI cardiac function in mice, likely via increases in SCFAs [88]. *Akkermansia* might be another promising candidate to test in CKD after being shown to alleviate inflammation and vascular calcification via increases in SCFAs in atherosclerotic rats [99]. The mediating effect of SCFAs and the aforementioned beneficial effects on cardiovascular health lead to the assumption that SCFA postbiotic supplementation could also form a promising approach in CKD that remains to be tested. While all these approaches try to improve dysbiosis or mimic features of beneficial microbiota, they remain relatively circumscribed. FMT may form a more holistic approach but despite successful use for treatment of antibiotic-resistant *Clostridioides difficile* infection, guidelines for safe and effective procedure, donor selection, and recipient requirements are still lacking [108]. Patients with high susceptibility to infection and *leaky gut*—both applicable to advanced CKD—may particularly be at risk after FMT. Of note, FMT induced tolerance in patients with graft-versus-host disease after allogenic stem cell transplantation most likely via increased Treg levels, highlighting the great potential of FMT, but the low number of patients studied warrants the evaluation of potential risks at a larger scale [109, 110].

The efficacy of anti-inflammatory therapy to lower CVD burden in CKD was demonstrated by the CANTOS trial. However, canakinumab could not reduce CVD to the general population level, and hence, studies on other anti-inflammatory treatment approaches may result in more effective strategies. Current studies in patients with CVD, but not specifically CKD, include treatment with anti-inflammatory low-dose IL-2 in patients with coronary artery disease analysing vascular inflammation and immune phenotype (IVORY trial) [111, 112]. Anti-inflammatory treatment with either cytokine or antibody administration imply a high cost at the current state and the subcutaneous administration route may limit patient adherence in comparison to oral administration of -biotics. While the latter may be addressed by use of small molecules instead of cytokines/antibodies, the high cost of currently tested anti-inflammatory drugs is unquestionable and application to a large portion of CVD patients would imply major costs for the healthcare system. Hence, economical aspects may favor -biotics instead of anti-inflammatory treatment and one may also speculate if broader effects of -biotics might be more effective than one single cytokine; however, this will have to be clarified in clinical trials. Moreover, it should be taken into account that both microbiome and inflammatory status are highly individual even in diseased patients. Consequently, precise microbiota, metabolomic, immune and inflammation profiling may confer potential to design therapeutic approaches in a more individual fashion—often referred to as personalized medicine—instead of ‘one-fits-all’ concepts, ultimately increasing treatment efficacy whilst reducing potential side effects.

## CONCLUSIONS

A growing body of research examined the role of microbiome-driven inflammation in CKD and led to the discovery of key features of microbiome dysbiosis, metabolic alterations and subsequent inflammatory mechanisms driving CVD. It is becoming more and more clear that changes in the gut microbiome—culminating in dysbiosis—are an important driver of inflammation in CKD. The present findings already give rise to potential microbiome- and inflammation-related therapeutic targets that show high potential to actually lower inflammatory and subsequently CVD burden. Notwithstanding, from pharmaceutical development and subsequent clinical trials—with relevant cardiovascular readouts only achievable in multiple years—to actual widespread use of such additive therapy for patients with CKD, there is still a rocky road lying ahead. Beyond that, knowledge gaps on how dysbiosis actually develops in CKD and how CKD affects the microbiome will have to be filled in future studies. Such host effects on the microbiome may be mediated, amongst others, by the immune system as depicted by Fatkhullina *el al.* showing a profound impact of anti-inflammatory cytokines on the microbiome [97] while Tang *et al.* could demonstrate the significance of host disease for microbiota composition in CVD models, specifically experimental MI [88]. A limitation of current studies also lies in the potential impact of high volume yet heterogeneous drug intake on the microbiome in CKD as drug intake was shown to have a major influence on the microbiome that might even exceed the effect of the disease itself [113]. While microbiome effects on the host in CKD are becoming increasingly clear, mechanisms of host-to-microbiome communication and implications of drug intake remain largely elusive in CKD and will have to be considered in future research to pave the way towards specific microbiome- and inflammation-targeted therapeutics, combatting the high cardiovascular burden from which patients with CKD suffer.

## Data Availability

No new data were generated or analysed in support of this research.
